# Structural Characterization of Core Region in *Erwinia amylovora* Lipopolysaccharide

**DOI:** 10.3390/ijms18030559

**Published:** 2017-03-04

**Authors:** Angela Casillo, Marcello Ziaco, Buko Lindner, Susana Merino, Elena Mendoza-Barberá, Juan M. Tomás, Maria Michela Corsaro

**Affiliations:** 1 Department of Chemical Sciences, University of Naples “Federico II”, Complesso Universitario Monte S. Angelo, Via Cintia 4, 80126 Naples, Italy; angela.casillo@unina.it (A.C.); marcello.ziaco@unina.it (M.Z.); 2 Division of Bioanalytical Chemistry, Research Center Borstel, Leibniz-Center for Medicine and Biosciences, Parkallee 10, D-23845 Borstel, Germany; blindner@fz-borstel.de; 3 Department of Genética, Microbiología y Estadística, Universidad de Barcelona, Diagonal 643, 08071 Barcelona, Spain; smerino@ub.edu (S.M.); elenademendoza@hotmail.com (E.M.-B.); jtomas@ub.edu (J.M.T.)

**Keywords:** lipopolysaccharide, *Erwinia amylovora*, NMR, ESI FT-ICR, structural determination

## Abstract

*Erwinia amylovora* (*E. amylovora*) is the first bacterial plant pathogen described and demonstrated to cause fire blight, a devastating plant disease affecting a wide range of species including a wide variety of *Rosaceae*. In this study, we reported the lipopolysaccharide (LPS) core structure from *E. amylovora* strain CFBP1430, the first one for an *E. amylovora* highly pathogenic strain. The chemical characterization was performed on the mutants *waaL* (lacking only the O-antigen LPS with a complete LPS-core), *wabH* and *wabG* (outer-LPS core mutants). The LPSs were isolated from dry cells and analyzed by means of chemical and spectroscopic methods. In particular, they were subjected to a mild acid hydrolysis and/or a hydrazinolysis and investigated in detail by one and two dimensional Nuclear Magnetic Resonance (NMR) spectroscopy and ElectroSpray Ionization Fourier Transform-Ion Cyclotron Resonance (ESI FT-ICR) mass spectrometry.

## 1. Introduction

*Erwinia amylovora* is the causal agent of fire blight, a disease of nutritionally important members of the family Rosaceae, such as apple and pear trees. The symptoms in apple plants are present on rootstocks, blossoms, shoots, and fruits [1]. On fruits, the disease provokes the developent of ooze, which is composed of bacteria, polysaccharides, and plant sap [1]. Bacteria are transported by insects, rain, birds, wind-wipping, and hail from the ooze to flowers. From flower infection, it can also be transferred to lateral parts of the plant through an endophytic mechanism [2].

Two major virulence determinants are known for the pathogenesis of *Erwinia*: (i) one involves the *hrp*/*dsp* gene cluster, the role of which is to secrete and deliver proteins from bacteria to plant apoplasts and cytoplasm [1]; (ii) the second is the production of two types of exopolysaccharides (EPS), amylovoran and levan [3,4].

The outer membrane (OM) of almost all Gram-negative bacteria and of some cyanobacteria [5,6,7,8] contains lipopolysaccharides (LPSs), where they constitute approximately 75% of the outer surface. They are amphiphilic endotoxic molecules necessary for the viability and survival of Gram-negative bacteria, as they seriously contribute to the structural integrity of the OM and to the protection of the bacterial cell envelope [9].

The colony morphology of Gram-negative bacteria can appear as smooth or rough as a consequence of a different structure of the lipopolysaccharides, named smooth (S-LPS) or rough (R-LPS), respectively. The structure of a S-LPS molecule can be described as three covalently linked domains: the glycolipid portion, called lipid A; the intermediate core oligosaccharide region (core); and the O-specific polysaccharide (O-chain) [10]. Instead, the R-LPSs (named lipooligosaccharides, LOSs) completely lacks the O-specific polysaccharide chain, either due to genetic mutation or to the inherent nature of bacteria [11].

Bacterial lipopolysaccharides show multiple roles in plant–microbe interactions. LPS give a contribution to the low permeability of the outer membrane, which acts as a barrier to protect microorganisms from plant-derived antimicrobial substances. LPS-defective mutants display augmented in vitro sensitivity to antibiotics and antimicrobial peptides and, upon introduction into susceptible plants, the numbers of viable bacteria often decay very quickly [12,13,14].

In this study we characterized the lipopolysaccharide core structure from the strain CFBP1430, the first one completely sequenced for a highly pathogenic *E. amylovora*. In particular, the results about *waaL*, *wabH*, and *wabG* mutants are reported. We studied *wabH* and *wabG* mutants because we found these genes in the *Erwinia amylovora* strain CFBP1430 *wb*; they were fully characterized in *Klebsiella pneumoniae* and *Serratia marcescens*, and both correspond to the outer-LPS core [15]. The lipooligosaccharides were degraded both by mild hydrazinolysis (*O*-deacylation) and hot KOH (*N*-deacylation). Both products were investigated by chemical analysis, by ^1^H and ^13^C NMR spectroscopy, and by ESI FT-ICR spectrometry.

## 2. Results and Discussion

### 2.1. Preparation and Structural Characterization of Oligosaccharides from EaΔwaaL LPS

The LPSs from the *waaL*, *wabH*, and *wabG* mutants (EaΔwaaL, EawabH, and EaΔwabG, respectively) of the *Erwinia amylovora* strain CFBP1430 were extracted by the phenol-chloroform-light petroleum (PCP) method. WabG is responsible for the transfer of d-GalA to the O-3 position of l,d-Hep II, and WabH transfers a d-GlcNAc residue from UDP-GlcNAc to the d-GalA [15]. The monosaccharides composition was performed as already reported [16]. In particular, the Gas Chromatography-Mass Spectrometry (GC-MS) analysis of methyl and octyl glycosides for EaΔwaaL and EaΔwabH mutant LPSs revealed the following sugars; d-Glc, d-GalA, d-GlcN, l,d-Hep, d,d-Hep, and Kdo. When this analysis was performed on EaΔwabG LPS, the lack of d-GalA and d,d-Hep was observed.

The removal of fatty acids from Ea∆waaL LPS was performed both by strong alkaline and acetic acid hydrolyses. The obtained products were analyzed by ESI FT-ICR mass spectrometry. In addition, mono- and two-dimensional NMR spectroscopy (^1^H,^1^H DQF-COSY, ^1^H,^1^H TOCSY, ^1^H,^1^H ROESY, ^1^H,^13^C HSQC-DEPT, ^1^H,^13^C HSQC-TOCSY, and ^1^H,^13^C HMBC) was allowed to assign all the proton and carbon chemical shifts of each mutant. The anomeric configurations were identified on the basis of both ^13^C chemical shift values and ^3^*J*_H1,H2_ coupling constants. The sequence of the residues in all the oligosaccharides was obtained both by long-range scalar couplings and by nuclear Overhauser enhancement (NOE) data.

**Scheme 1:**
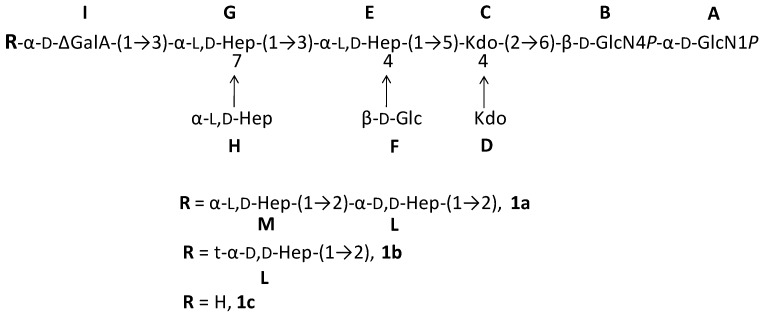
Structures of the totally deacylated LPS fraction (L-OS_KOH_) isolated from the *E. amylowora* EaΔwaaL mutant.

**Figure 1:**
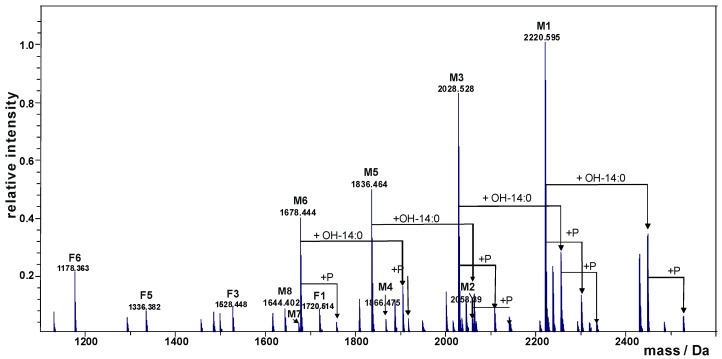
Charge deconvoluted ElectroSpray Ionization Fourier Transform-Ion Cyclotron Resonance (ESI FT-ICR) mass spectrum of the totally deacylated lipopolysaccharide (LPS) fraction (L-OS_KOH_) isolated from the *E. amylowora* EaΔwaaL mutant. The spectrum was acquired in negative ion mode. The mass numbers given refer to the monoisotopic peak of the neutral molecular species.

Starting from the totally deacylated product (L-OS_KOH_, Scheme 1, structures **1a**–**c**), the negative ion mode ESI FT-ICR mass spectrum of the sample identified eight main species (Figure 1 and Table 1). Species **M1**, occurring at 2220.595 Da (calculated molecular mass 2220.586 Da), represented the higher molecular mass oligosaccharide chain containing the phosphorylated lipid A backbone and the core structure.

In particular, the following composition was attributed to the **M1** species: GlcN_2_P_2_Kdo_2_Hep_5_Glc_1_ΔGalA, where ΔGalA represents an unsaturated galacturonic acid. The presence of this acid unit in the **M1** species clearly indicated that the strong alkaline conditions determined the lack of one or more sugars from GalA, due to a β-elimination reaction [17]. Species **M3**, **M5**, and **M8** differed from **M1** in their heptoses composition, displaying four units in **M3** and three and two in **M5** and **M8**, respectively. The remaining species, **M2**, **M4**, and **M7**, lacked a glucose unit respect to **M1**, **M3**, and **M5**, respectively, thus confirming the terminal position for this residue, while the **M6** species lacked ΔGalA compared to **M5**. Further mass peaks originate from an additional phosphate group P (Δm = +79.966 u) and/or one linked C14:0(3OH) fatty acid (Δm = +226.193 u) due to incomplete deacylation. Finally, **F1**–**F6** are fragments induced during the ESI process leading to the cleavage of the lipid A backbone GlcN_2_P_2_ (Δm = −500.081 u).

**Figure 2:**
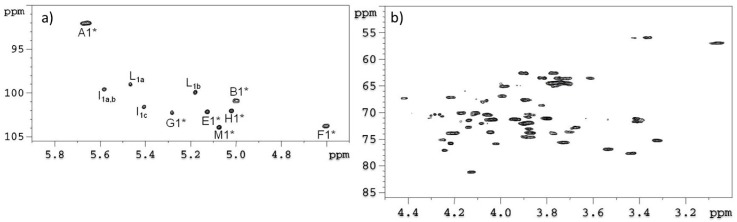
Anomeric (**a**) and carbinolic (**b**) region of ^1^H-^13^C HSQC-DEPT of the totally deacylated LPS (L-OS_KOH_) from *E. amylowora waaL* mutant. All the correlation cross-peaks in the anomeric region indicate the signals of structures **1a**–**c**. The spectrum was recorded in D_2_O at 298 K at 600 MHz.

Despite the complexity of the ^1^H-^13^C Heteronuclear Single Quantum Coherence-Distortionless Enhancement by Polarization Transfer (HSQC-DEPT) experiment (Figure 2), it was possible to assign the signals of the main species, **M1**, **M3**, and **M5**. All the NMR data (Table 2, structures **1a**–**c**) strongly suggested that the fractions L-OS_KOH_ and the structure of the oligosaccharide OS1 obtained from the previously published *K. pneumoniae* 52145 *waaL* mutant [17] possessed a common structural element constituted by the residues **A**, **B**, **C**, **D**, **E**, **F**, **G**, **H**, and **I** (monosaccharide units are as shown in Table 2), in agreement with the strain genomics.

All the species possessed α-GlcN1P residue at the reducing end (**A**, H1, δ 5.65 ppm; C1, δ 92.0 ppm), originating from the lipid A backbone. The second residue of glucosamine of the lipid A (**B**, H1, δ 4.99 ppm, C1, δ 100.9 ppm) was O-4 phosphorylated, as suggested by the downfield shift of both H4 and C4 chemical shifts [18]. In all the oligosaccharides corresponding to the **M1**–**M7** species, the core region was linked to the lipid A backbone through an α-Kdo residue **C**, which was substituted at O-4 position by a second residue of Kdo (**D**).

Residues **E**, **G**, **H**, **L**, and **M** were identified as five *manno*-configured α-heptopyranoses due to their small coupling constants ^3^*J*_H1,H2_ and ^3^*J*_H2,H3_ values. Downfield shifted carbon signals with respect to reference values [19] indicated substitutions at O-3 and O-4 of residue **E** (C3 at 77.1 and C4 at 75.1 ppm, respectively) and at O-3 and O-7 of residue **G** (C3 at 81.1 ppm and C7 at 71.2 ppm). Both residues **H** and **M** were identified as terminal units as none of their carbon signals were downfield shifted. All the heptose residues were found to be l,d-configured except for **L**; in fact, for this residue, the chemical shift of its C6 at 72.1 ppm suggested a d,d-configuration [20]. In addition, the downfield shift of its C2 resonance at 80.0 ppm clearly indicated that it was substituted at this position. Residue **I**, with H1/C1 at 5.58/99.6 ppm and H4/C4 at 5.82/108.9 ppm, was identified as a α-threo-hex-4-enuronopyranosyl unit (ΔGalA).

Finally, residue **F** was assigned to a β-glucose residue, on the basis of the proton multiplicities obtained by the DQF-COSY and TOCSY experiments. No downfield carbon chemical shifts were observed for this residue, thus indicating its terminal non reducing end position.

The sequence of the residues was deduced from the HMBC experiment, which showed correlations between C1 of **L** and H2 of **I**, H1 of **I** and C3 of **G**, H1 of **G** and C3 of **E**, C1 of **H** and H7 of **G**, and C1 of **F** and H4 of **E**.

In addition, residue **M** was found to be linked at the O-2 position of residue **L**, as shown by NOE contacts among H1 of **M** and both H1 and H2 of **L**, observed in the ROESY experiment. This last completed and confirmed the above sequence by inter-residual dipolar couplings. In the same spectrum, the intra-residue NOE contacts were in agreement with the assigned relative configuration of the monosaccharides.

All these data indicated for **M1** species of L-OS_KOH_ the oligosaccharide structure **1a**. Structures **1b** and **1c**, corresponding to the species **M3** and **M5**, were identified on the basis of their terminal residue d,d-heptose and ΔGalA, respectively.

**Scheme 2:**
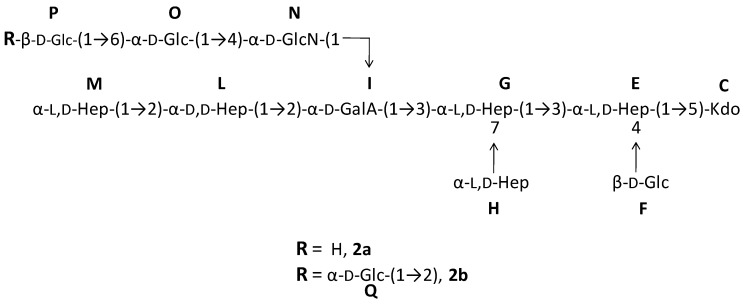
Structures of acetic acid hydrolysed LPS fraction (L-OS_AcOH_) isolated from the *E. amylowora* EaΔwaaL mutant.

The LPS of Ea∆waaL mutant was then treated with 5% acetic acid at 100 °C, and the supernatant was analyzed (L-OS_AcOH_, Scheme 2, structures **2a**,**b**). Gas Chromatography-Mass Spectrometry (GC-MS) analysis of the partially methylated alditol acetates of this sample indicated the presence of terminal l,d-heptose, terminal glucose, terminal d,d-heptose, 2-substituted d,d-heptose, 2-substituted glucose, 6-substituted glucose, 4-substituted glucosamine 3,4-substituted l,d-heptose, and 3,7-substituted l,d-heptose. Each sugar was recognized from both its Electronic Ionization (EI) mass spectrum and the GC column retention time, by comparison with standards. The Kdo was not revealed in this analysis, due to the presence of its anhydro form.

**Figure 3:**
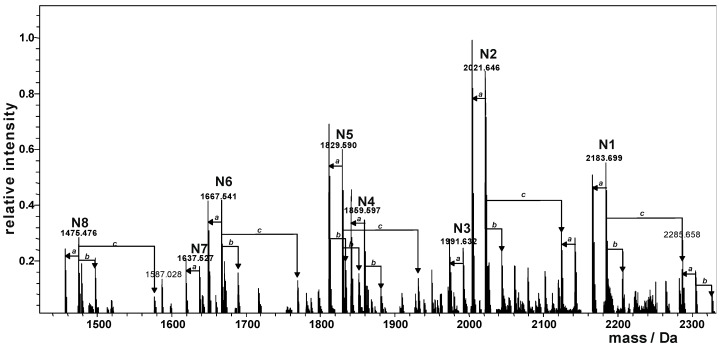
Charge deconvoluted ESI FT-ICR mass spectrum of supernatant of the acetic acid hydrolysed LPS fraction (L-OS_AcOH_) isolated from *E. amylowora waaL* mutant. The spectrum was acquired in negative ion mode. The mass numbers given refer to the monoisotopic peak of the neutral molecular species. Further mass peaks originate from a: loss of H_2_O (Δm = −18.010 u); b: sodium adducts [M − H + Na] (Δm = +21,979 u); c: an additional phosphate group (P − H + Na) (Δm = +101.948 u).

The negative mode ESI FT-ICR mass spectrum of **2** (Figure 3 and Table 3) and NMR data (Table 2) revealed a complex mixture of oligosaccharides. The main component of the mass spectrum **N2** species was revealed to possess a common part with the oligosaccharides of L-OS_KOH_, constituted by the residues **E**, **F**, **G**, **H**, **L**, and **M**. In addition, the trisaccharide β-Glc-(1 → 6)-α-Glc-(1 → 4)-α-GlcN-(1 → was present at position O-4 of α-GalA **I**.

We compared our NMR data with the data already published for deacylated oligosaccharides from *Serratia marcenscens* [19], and we found very similar chemical shifts, even if we recognized in *Erwinia* an additional minor component (**N1** species, Table 2 and Figure 3). In fact, the molecular mass of the **N1** species indicated an additional hexose with respect to that of **N2**. This residue (**Q**, Table 2) was identified as gluco-configurated with an α anomeric configuration, as suggested by its ^3^*J*_H1,H2_ anomeric coupling constant of 3.5 Hz. A long-range heteronuclear scalar coupling between H1 of **Q** and C2 of **P** indicated that in the **N1** species the oligosaccharide substituting the O-4 of GalA had the structure: α-Glc-(1 → 2)-β-Glc-(1 → 6)-α-Glc-(1 → 4)-α-GlcN. All these data were in agreement with the methylation analysis, except for the 2,4-disubstituted galacturonic acid residue, which was absent in the GC-MS chromatogram. This fact could be due to the hindrance of both oligosaccharides substituting O-2 and O-4 positions of GalA, thus preventing its methylation.

### 2.2. Preparation and Structural Characterization of Oligosaccharides from EaΔwabH and EaΔwabG LPSs

The LPSs from EaΔwabH and EaΔwabG mutants were completely deacylated by hydrazine followed by the KOH reaction. After purification on a gel filtration Sephadex G10 column, the samples, named H-OS_KOH_ and G-OS_KOH_ respectively, were analysed by mono- and two-dimensional NMR experiments (Table 2). The acetic acid hydrolysis was not performed on these two mutants as no β-degradation was observed for these samples, indicating that no information was lost.

The ^1^H NMR spectrum of the totally deacylated Ea∆wabH LPS (H-OS_KOH_, Figure S1, Supporting Information) showed nine main anomeric proton signals, assigned to residues **A**–**M**, in the range 5.6–4.4 ppm. Signals in the range 2.2–1.5 ppm, attributable to the presence of the Kdo units, were also present.

**Scheme 3:**
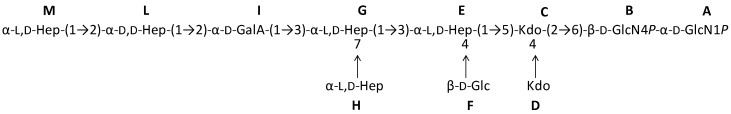
Structures of the totally deacylated LPS fraction (H-OS_KOH_) isolated from the *E. amylowora* Ea∆wabH mutant.

The galacturonic acid residue (residue **I**, Scheme 3, structure **3**) was recognized from the chemical shift of its C6 at δ 176.8 ppm (Table 2), which correlated with its H5 proton at δ 4.34 ppm in the Heteronuclear Multiple Bond Correlation (HMBC) experiment.

In addition, the O-substitution at position 2 of **I** was inferred by the long-range between H1 of **L** and C2 of **I**. The value of the coupling constant ^3^*J*_H1,H2_ of the anomeric proton was found to be 3.5 Hz, thus indicating for residue **I** an α configuration. The chemical shifts of heptoses **E**, **G**, **H**, **L**, and **M** were in agreement with that already found for structure **1**, as well as that of terminal β-Glc **F**. Finally, both the HMBC (Figure S2) and ROESY experiments indicated the sequence shown in structure **3**, which is in agreement with strain genomics.

**Scheme 4:**
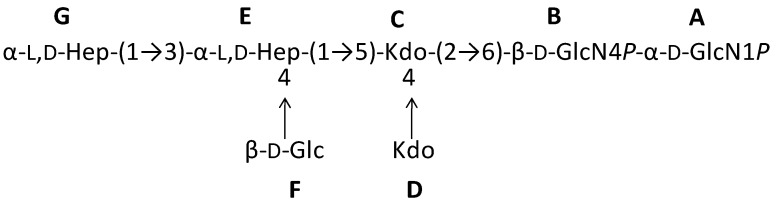
Structures of the totally deacylated LPS fraction (G-OS_KOH_) isolated from the *E. amylowora* Ea∆wabG mutant.

The ^1^H-NMR spectrum of the totally deacylated Ea∆wabG LPS, (G-OS_KOH_, Figure S3, Supporting Information) showed only five anomeric proton signals. All the ^1^H and ^13^C chemical shifts of each residue were identified and assigned by two-dimensional NMR experiments (Table 2). NMR data, together with glycosyl analysis, indicated the lack of the trisaccharide α-Hep-(1 → 2)-α-Hep-(1 → 2)-α-GalA. This was in agreement with the hypothesis of assignment of the *wabG* gene to a galacturonic acid residue glycosyltransferase. In addition, the ^1^H,^13^C HSQC-DEPT NMR experiment (Figure S4) revealed the absence of the inner core heptose residue **H**, since only two l,d-heptose anomeric signals, **E** and **G**, were found (Scheme 4, structure **4**).

This fact suggested that the lack of GalA residue may preclude the addition of heptose **H** to the position O-7 of heptose **G**.

## 3. Experimental Section

### 3.1. Bacteria Growth and LPS Isolation

Dried bacteria cells from EaΔwaaL (3.3 g), EaΔwabH (2.63 g), and EaΔwabG (3.58 g) mutants of *E. amylovora* strain CFBP1430 were all extracted by the PCP method [21] to give 208 mg of LPS (LPS_PCP_ yield 6.3% *w*/*w* of dried cells) for the *waaL* mutant, 124 mg of LPS for the *wabH* mutant (LPS_PCP_ yield 4.2% *w*/*w* of dried cells), and 170 mg of LPS (LPS_PCP_ yield 4.7% *w*/*w* of dried cells) for the *wabG* mutant, respectively.

### 3.2. Sugar Analysis

The sugar analysis was performed as reported [16]. The absolute configurations of the sugars were determined by gas chromatography of the acetylated (*S*)-2-octyl glycosides [22]. The derivatized samples were injected into an Agilent Technologies gas chromatograph 6850A equipped with a mass selective detector 5973N and a Zebron ZB-5 capillary column (Phenomenex, Bologna, Italy, 30 m × 0.25 mm i.d., flow rate 1 mL/min, He as carrier gas). The following temperature programs were used; 140 °C for 3 min, 140 °C → 240 °C at 3 °C/min (acetylated methyl glycosides), 150 °C for 5 min, 150 °C → 240 °C at 6 °C/min, and 240 °C for 5 min (acetylated octyl glycosides).

### 3.3. Mild Acid Hydrolysis

The LPS of the EaΔwaaL mutant (20 mg) was treated with 5% aqueous CH_3_COOH (2 mL, 100 °C for 4 h). After centrifugation (7500 rpm, 4 °C, 30 min), the pellet was washed twice with water. Then, the supernatant layers were pooled together and lyophilized. The sample was then fractionated on a Bio-Gel P-10 column (Biorad, 1.5 × 110 cm, flow rate 15 mL/h, fraction volume 2 mL) and eluted with water buffered with 0.05 M pyridine and 0.05 M AcOH. The fractions containing oligosaccharides were pooled and named L-OS_AcOH_ (10.1 mg).

### 3.4. Deacylation of the LOSs

The LOS from each mutant (100 mg) was dried over phosphorus anhydride under a vacuum and treated with hydrazine (5 mL, at 37 °C for 2 h) [23]. Cold acetone was added, and the pellet was recovered after centrifugation at 4 °C and 7000 rpm for 30 min. After being washed three times with acetone, it was suspended in water and lyophilized, obtaining L_LOS-OH_ (68.6 mg), H_LOS-OH_ (72.4 mg), and G_LOS-OH_ (65.9 mg).

The partially deacylated LPS (LOS-OH) from each mutant was dissolved in KOH 4 M and incubated at 120 °C for 16 h. The KOH was neutralized with HCl, and the mixture was extracted three times with CHCl_3_. The aqueous phases were recovered and desalted on a Sephadex G-10 column (Amersham Biosciences, Little Chalfont, UK, 2.5 × 43 cm, 31 mL·h^−1^, fraction volume 2.5 mL, eluent NH_4_HCO_3_ 10 mM). The eluted oligosaccharides mixture was then lyophilized (L-OS_KOH_ 19 mg; H-OS_KOH_ 13 mg; G-OS_KOH_ 20 mg).

### 3.5. Methylation Analysis

The linkage positions of the monosaccharides were determined by GC-MS analysis of the partially methylated alditol acetates (PMAAs). The acetic acid oligosaccharides fraction of EaΔwaaL mutant (1 mg) was first reduced with NaBD_4_ and then methylated with CH_3_I (100 µL) and NaOH powder in dimethyl sulfoxide (DMSO) (300 µL) for 20 h [24].

The reduction of the carboxymethyl groups was obtained by treating the sample with sodium boro deuteride (NaBD_4_). After the reaction working up, the sample was totally hydrolyzed with 2 M trifuoroacetic acid (TFA) at 120 °C for 2 h, reduced again with NaBD_4_, and acetylated with Ac_2_O and pyridine (50 µL each, 100 °C for 30 min) [25]. The PMAA mixture was analyzed by GC-MS with the following temperature program: 90 °C for 1 min, 90 → 140 °C at 25 °C/min, 140 °C → 200 °C at 5 °C/min, 200 → 280 °C at 10 °C/min, and 280 °C for 10 min.

### 3.6. Mass Spectrometry Analysis

Mass spectra were (ESI FT-ICR) were performed in negative ion mode using an APEX QE (Bruker Daltonics GmbH, Bremen, Germany) equipped with a 7 Tesla actively shielded magnet. The sample concentration was ~10 ng/µL. The solutions were sprayed at a flow rate of 2 µL/min and analyzed. The mass spectra obtained were charge-deconvoluted, and the mass numbers reported refer to the monoisotopic masses of the neutral molecules.

### 3.7. NMR Spectroscopy

^1^H and ^13^C NMR spectra were performed using a Bruker Avance 600 MHz spectrometer (Milano, Italy) equipped with a cryoprobe. All 2D homo- and heteronuclear experiments (double quantum-filtered correlation spectroscopy, DQF-COSY; total correlation spectroscopy TOCSY; rotating-frame nuclear Overhauser enhancement spectroscopy, ROESY; nuclear Overhauser effect spectroscopy, ^1^H,^13^C HSQC-DEPT; and heteronuclear multiple bond correlation, ^1^H,^13^C HMBC) were obtained using the standard pulse sequences available in the Bruker software (TopSpin 3.1 version). The TOCSY and ROESY experiments were obtained with mixing times of 100 ms. Chemical shifts were measured at 298 K in D_2_O.

## 4. Conclusions

**Scheme 5:**
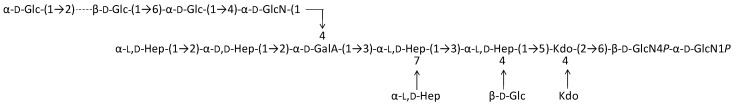
Core oligosaccharide structures from *E. amylowora* strain CFBP1430 LPS.

In this work, for the first time, we have characterised the complete structure of the core oligosaccharide from *E. amylowora* strain CFBP1430 LPS (Scheme 5, structure **5**). To this aim, we prepared three mutants, i.e. *waaL*, *wabH*, and *wabG*, the purified lipopolysaccharides of which were characterised. The reported data showed that the core oligosaccharides here reported share structural fragments with those of *Klebsiella pneumoniae* and *Serratia marcescens*. In particular, by comparison with the core of *S. marcescens* the present structure lacks the non-stoichiometric Ko residue, a feature considered useful to distinguish between the genera *Burkholderia* and *Pseudomonas*.

## Figures and Tables

**Table 1:** Composition of the main species observed in the charge deconvoluted ESI FT-ICR mass spectrum of the totally deacylated LPS (L-OS_KOH_) from a *E. amylowora waaL* mutant (EaΔwaaL).

Species	Observed Mass (Da)	Calculated Mass (Da)	Composition
**M1**	2220.595	2220.589	GlcN_2_P_2_Kdo_2_Hep_5_Glc_1_ΔHexA
**M2**	2058.390	2058.536	GlcN_2_P_2_Kdo_2_Hep_5_ΔHexA
**M3**	2028.528	2028.525	GlcN_2_P_2_Kdo_2_Hep_4_Glc_1_ΔHexA
**M4**	1866.475	1866.473	GlcN_2_P_2_Kdo_2_Hep_4_ΔHexA
**M5**	1836.464	1836.462	GlcN_2_P_2_Kdo_2_Hep_3_Glc_1_ΔHexA
**M6**	1678.444	1678.440	GlcN_2_P_2_Kdo_2_Hep_3_Glc_1_
**M7**	1674.410	1674.409	GlcN_2_P_2_Kdo_2_Hep_3_ΔHexA
**M8**	1644.402	1644.399	GlcN_2_P_2_Kdo_2_Hep_2_Glc_1_ΔHexA

**Table 2:** ^1^H and ^13^C chemical shifts (δ) of sugar residues of core oligosaccharides from *Erwinia amylovora* LPS. Nuclear Magnetic Resonance (NMR) spectra were recorded in D_2_O at 600 MHz at 298 K.

Residue	Nucleus	H1	H2	H3	H4	H5	H6	H7	H8
		C1	C2	C3	C4	C5	C6	C7	C8
α-GlcN1*P* A, 1a–c	^1^H	5.65	3.37	3.94	3.41	4.21	3.88/4.27		
	^13^C	92.0	55.9	71.2	71.1	73.8	70.2		
α-GlcN1*P* A, 3	^1^H	5.53	3.28	3.80	3.51	4.05	3.65/4.20		
	^13^C	92.0	55.9	71.0	70.9	73.4	71.0		
α-GlcN1*P* A,4	^1^H	5.54	3.30	3.80	3.51	4.04	3.65/4.19		
	^13^C	92.0	57.1	71.0	71.1	73.6	71.3		
β-GlcN4*P* B, 1a–c	^1^H	4.99	3.06	3.88	3.88	3.72	3.62/3.75		
	^13^C	100.9	56.9	73.1	74.6	75.6	63.5		
β-GlcN4*P* B, 3	^1^H	4.72	2.93	3.73	3.72	3.60	3.35/3.57		
	^13^C	100.9	56.9	70.9	74.0	72.3	63.7		
β-GlcN4*P* B, 4	^1^H	4.73	2.94	3.72	3.73	3.63	3.35/3.60		
	^13^C	100.9	57.1	70.9	73.8	75.3	64.0		
4,5-Kdo C, 1a-c	^1^H	n.d.	n.d.	1.88/2.12	4.21	4.21	4.14	4.17	3.77/3.92
	^13^C			36.0	73.9	75.7	72.9	70.2	64.9
4,5-Kdo C, 3	^1^H	175.6	100.7	1.81/1.98	4.03	4.17	3.83	3.51	3.62-3.87
	^13^C			36.0	71.6	70.1	74.0	73.4	64.3
4,5-Kdo C, 4	^1^H	176.3	101.1	1.88/2.04	3.99	4.17	3.88	3.54	3.61-3.87
	^13^C			36.2	72.0	70.4	71.9	73.6	64.7
t-Kdo D, 1a-c	^1^H	n.d.	n.d.	1.82/2.18	4.09	4.06	3.71	4.09	3.77/3.99
	^13^C			35.9	68.1	67.7	73.7	72.1	65.0
t-Kdo D, 3	^1^H	176.1	102.6	1.67/2.06	3.95	3.95	3.60	3.56	3.56/3.87
	^13^C			35.7	67.4	67.4	73.9	73.1	64.5
t-Kdo D, 4	^1^H	176.1	102.8	1.66/2.00	3.96	3.94	3.62	3.56	3.61/3.78
	^13^C			36.3	67.7	67.9	73.3	73.3	65.1
3,4-α-l,d-Hep E, 1a–c	^1^H	5.12	4.13	4.24	4.25	4.10	4.17	3.76	
	^13^C	102.2	71.5	77.1	75.1	70.1	70.2	64.5	
3,4-α-l,d-Hep E, 2a,b	^1^H	5.12	4.07	4.23	4.23	4.10	4.15	3.75	
	^13^C	101.9	71.4	77.1	75.3	70.3	69.8	64.4	
3,4-α-l,d-Hep E, 3	^1^H	5.19	3.95	4.06	4.19	4.18	4.00	3.69	
	^13^C	100.1	71.8	75.1	74.4	72.2	70.2	65.1	
3,4-α-l,d-Hep E, 4	^1^H	5.16	4.00	3.99	4.15	4.12	3.98	3.68/3.89	
	^13^C	103.0	72.0	75.6	74.6	73.3	70.3	73.3	
t-β-Glc F, 1a-c	^1^H	4.60	3.33	3.53	3.41	3.42	3.77/3.90		
	^13^C	103.8	75.3	77.0	71.7	77.7	62.5		
t-β-Glc F, 2a,b	^1^H	4.62	3.31	3.54	3.41	3.42	3.77/3.90		
	^13^C	104.0	75.3	77.0	71.1	77.5	62.5		
t-β-Glc F, 3	^1^H	4.46	3.18	3.41	3.26	3.30	3.63/3.78		
	^13^C	103.2	75.0	76.5	71.1	77.3	62.7		
t-β-Glc F, 4	^1^H	4.43	3.19	3.39	3.23	3.36	3.61-3.85		
	^13^C	103.6	75.0	76.9	71.6	77.8	62.8		
3,7-α-l,d-Hep G, 1a–c	^1^H	5.28	4.26	4.13	3.99	3.78	4.21	3.80	
	^13^C	102.1	70.8	81.1	66.9	74.0	67.1	71.2	
3,7-α-l,d-Hep G, 2a,b	^1^H	5.26	4.12	4.03	4.01	3.90	4.17	3.77	
	^13^C	102.0	71.1	80.8	66.9	74.0	71.1	71.2	
3,7-α-l,d-Hep G, 3	^1^H	5.27	4.08	3.89	3.83	3.92	n.d.	3.69	
	^13^C	102.2	70.7	80.6	66.9	73.3		72.7	
t-α-l,d-Hep G, 4	^1^H	5.16	4.06	3.77	3.77	3.56	3.95	3.55	
	^13^C	100.1	71.0	72.0	67.6	73.2	70.3	64.7	
t-α-l,d-Hep H, 1a–c	^1^H	5.02	4.04	3.90	3.89	3.67	4.06	3.72	
	^13^C	102.0	73.6	70.9	67.6	72.9	70.5	63.6	
t-α-l,d-Hep H, 2a,b	^1^H	4.94	4.02	4.02	3.87	3.64	n.d	n.d	
	^13^C	101.8	73.1	71.3	67.6	72.7			
t-α-l,d-Hep H, 3	^1^H	4.84	3.88	3.91	3.88	3.63	3.93	3.64	
	^13^C	102.9	71.3	70.3	67.0	73.4	64.6	62.9	
2-Δ-GalA I, 1a,b	^1^H	5.58	4.02	4.53	5.82				
	^13^C	99.6	75.8	65.8	108.9				
t-Δ-GalA I, 1c	^1^H	5.40	3.87	4.42	n.d.				
	^13^C	101.6	71.9	67.3					
2,4-α-GalA I, 2a,b	^1^H	5.48	4.07	4.22	4.23	4.47			
	^13^C	99.5	80.4	68.5	77.2	72.6	176.2		
2-α-GalA I, 3	^1^H	5.39	3.91	3.96	4.21	4.34			
	^13^C	98.9	80.6	69.2	72.2	73.7	176.8		
2-α-d,d-Hep L, 1a	^1^H	5.35	4.06	3.97	3.89	n.d.	4.12	3.61	
	^13^C	98.9	80.0	71.4	68.5	n.d.	72.1	63.5	
t-α-d,d-Hep L, 1b	^1^H	5.18	4.03	3.90	3.83	3.87	4.04	3.82	
	^13^C	99.9	71.3	72.1	68.7	73.9	71.4	63.5	
2-α-d,d-Hep L, 2a,b	^1^H	5.28	3.99	3.99	3.81	3.99	4.05	3.84	
	^13^C	96.9	80.8	71.4	68.9	75.0	71.4	63.6	
2-α-d,d-Hep L, 3	^1^H	4.99	3.87	3.75	3.67	3.92	4.09	3.69	
	^13^C	98.3	80.6	71.7	68.7	73.3	73.1	63.1	
t-α-l,d-Hep M, 1a	^1^H	5.09	4.07	4.07	3.88	3.70	n.d.	n.d.	
	^13^C	103.5	71.3	73.7	67.7	73.1			
t-α-l,d-Hep M, 2a,b	^1^H	5.05	4.05	4.06	3.86	n.d.	n.d.	n.d.	
	^13^C	103.5	71.4	73.8	67.7				
t-α-l,d-Hep M, 3	^1^H	5.21	3.89	3.75	3.83	4.18	n.d.	3.56/3.78	
	^13^C	101.9	71.5	70.1	66.8	72.1		64.8	
4-α-GlcN N, 2a,b	^1^H	5.19	3.27	3.85	3.79	4.17	3.83		
	^13^C	97.6	55.3	72.5	77.0	72.5	61.3		
6-α-Glc O, 2a,b	^1^H	5.49	3.62	3.74	3.90	3.54	3.88/4.20		
	^13^C	100.7	72.8	74.2	73.0	70.8	69.8		
t-β-Glc P, 2a	^1^H	4.50	3.33	3.51	3.42	3.47	3.74/3.92		
	^13^C	103.9	74.5	77.0	71.0	77.3	62.3		
2-β-Glc P, 2b	^1^H	4.62	4.47	3.59	3.45	3.46	3.74/3.92		
	^13^C	103.9	78.9	75.9	70.9	77.3	62.3		
t-β-Glc Q, 2b	^1^H	4.36	3.61	3.75	3.47	4.17	n.d.		
	^13^C	99.2	72.9	74.2	70.8	72.9			

**Table 3:** Composition of the main species observed in the charge deconvoluted ESI FT-ICR mass spectrum of supernatant of the acetic acid hydrolysed LPS fraction (L-OS_AcOH_) isolated from *E. amylowora waa* L. mutant.

Species	Observed Mass (Da)	Calculated Mass (Da)	Composition
**N1**	2183.699	2183.698	Kdo_1_Hep_5_Glc_4_GalA_1_GlcN_1_
**N2**	2021.646	2021.645	Kdo_1_Hep_5_Glc_3_GalA_1_GlcN_1_
**N3**	1991.632	1991.635	Kdo_1_Hep_4_Glc_4_GalA_1_GlcN_1_
**N4**	1859.597	1859.592	Kdo_1_Hep_5_Glc_2_GalA_1_GlcN_1_
**N5**	1829.590	1829.578	Kdo_1_Hep_4_Glc_3_GalA_1_GlcN_1_
**N6**	1667.541	1667.529	Kdo_1_Hep_4_Glc_2_GalA_1_GlcN_1_
**N7**	1637.527	1637.518	Kdo_1_Hep_3_Glc_3_GalA_1_GlcN_1_
**N8**	1475.476	1475.466	Kdo_1_Hep_3_Glc_2_GalA_1_GlcN_1_

## Supplementary Materials

Supplementary materials can be found at www.mdpi.com/1422-0067/18/3/559/s1.
