# Supplementary material for: Structural Characterization of Core Region in Erwinia amylovora Lipopolysaccharide
Source: Int J Mol Sci. 2017 Mar 4;18(3):559. doi: 10.3390/ijms18030559 (PMC5372575; doi:10.3390/ijms18030559)
Supplement: Supplementary file 1 [file ijms-18-00559-s001.pdf]

Supplementary Materials: Structural Characterization of Core Region in *Erwinia amylovora* Lipopolysaccharide

Angela Casillo, Marcello Ziaco, Buko Lindner, Susana Merino, Elena Mendoza-Barberá, Juan M. Tomás and Maria Michela Corsaro

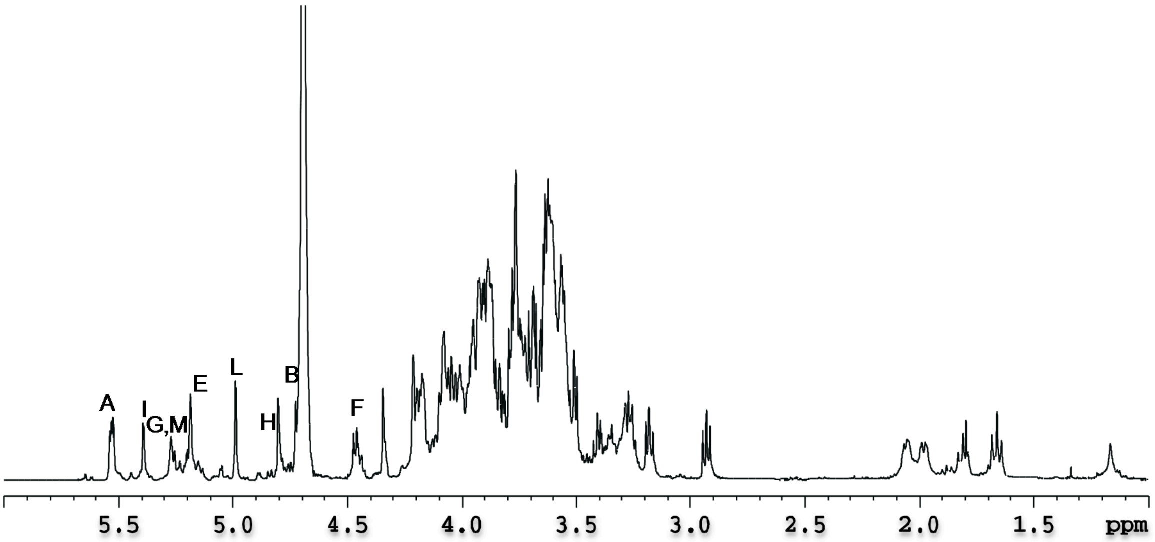

Figure S1. ^{1}H NMR spectrum from the totally deacylated LPS (H-OS$_{KOH}$) from *E. amylowora wabH* mutant. The spectrum was recorded in D_2O at 298 K at 600 MHz.

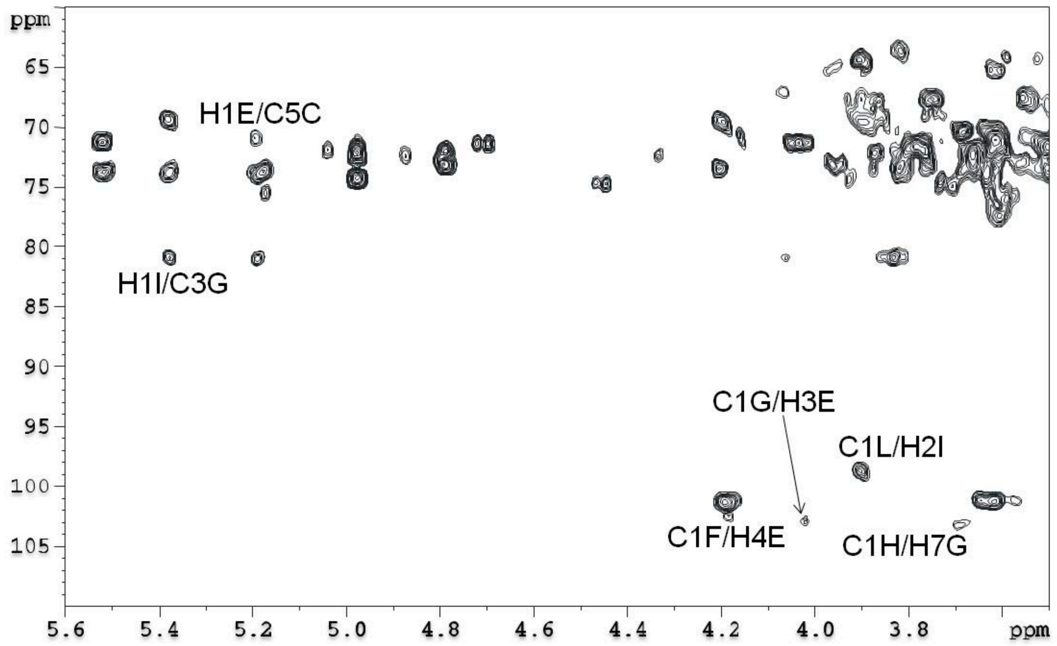

Figure S2. Relevant section of HMBC spectrum of the totally deacylated LPS (H-OS$_{KOH}$) from *E. amylowora wabH* mutant. The spectrum was recorded in D_2O at 298 K at 600 MHz.

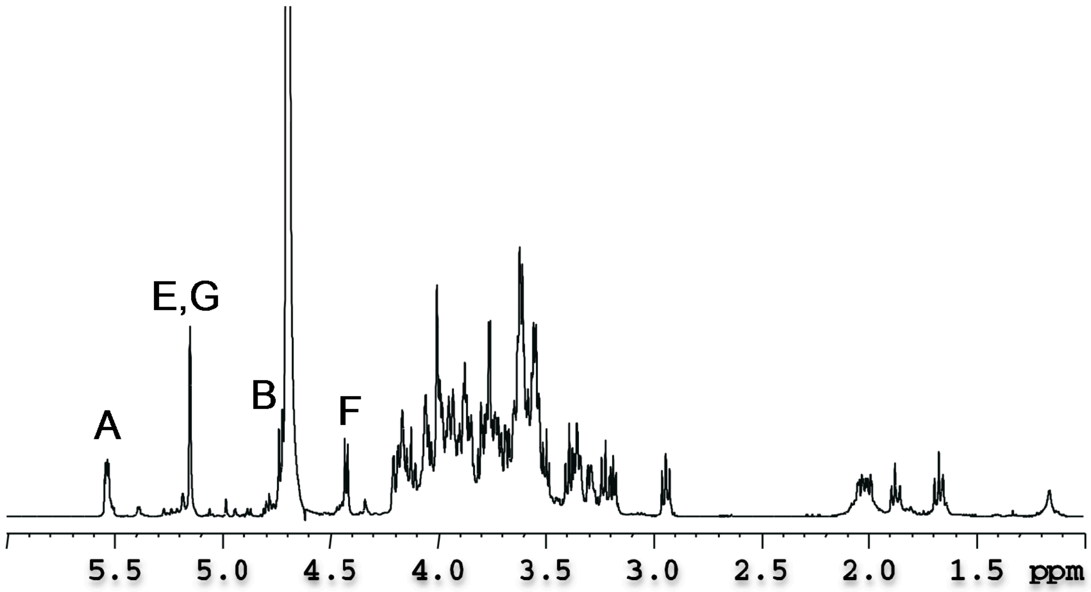

Figure S3. ^{1}H NMR spectrum from the totally deacylated LPS (G-OS$_{KOH}$) from *E. amylowora wabG* mutant. The spectrum was recorded in D_2O at 298 K at 600 MHz.

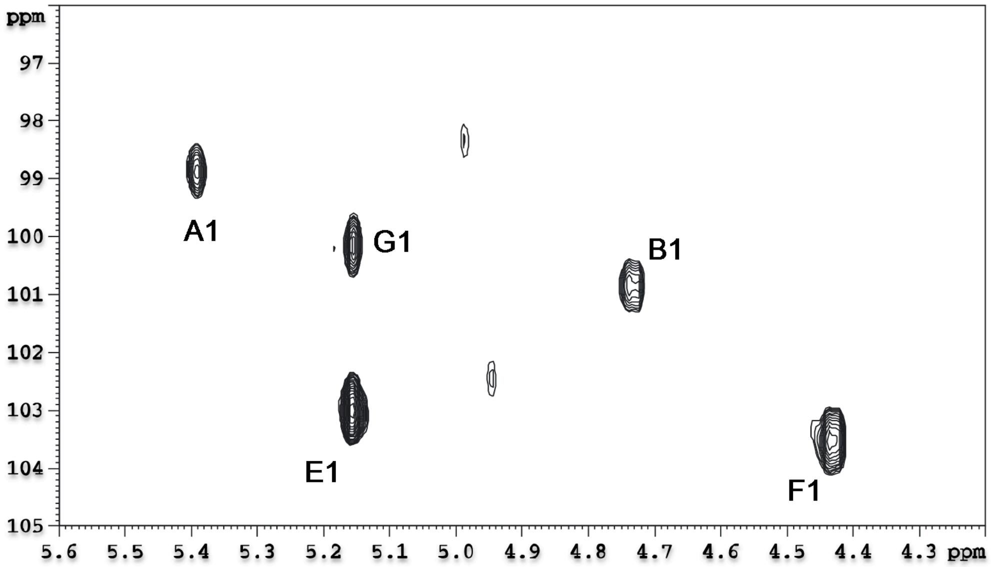

Figure S4. Anomeric region of ^{1}H-^{13}C HSQC-DEPT of the totally deacylated LPS (G-OS$_{KOH}$) from *E. amylowora wabG* mutant. The spectrum was recorded in D_2O at 298 K at 600 MHz.
